# In-Hospital Outcomes of Bariatric Surgery in People Living with HIV: A Nationwide Analysis

**DOI:** 10.1007/s11695-025-08007-z

**Published:** 2025-07-09

**Authors:** Leandro Sierra, Varun Aitharaju, Renan Prado, Michael Cymbal, Arjun Chaterjee, Akash Khurana, Roma Patel, Stephen Firkins, Roberto Simons-Linares

**Affiliations:** 1 Department of Medicine, Cleveland Clinic Foundation, Cleveland, OH USA; 2 Digestive Diseases and Surgery Institute, Cleveland Clinic Foundation, Cleveland, OH USA

**Keywords:** Bariatric Surgery, Human Immunodeficiency Virus, Peri-operative Care, Peri-operative Complications, Mortality Outcomes

## Abstract

**Background:**

Bariatric surgery is a growing treatment for Class 3 obesity in people with HIV, but concerns remain about antiretroviral therapy pharmacokinetics, comorbidities, and mortality. Existing studies confirm safety but are limited by small sample sizes and outdated cohorts. This study utilizes a recent, large U.S. inpatient cohort to address gaps.

**Methods:**

We performed a retrospective cohort study using the Nationwide Inpatient Sample from 2015 to 2021, identifying hospitalizations for bariatric surgery among patients with and without HIV. We compared demographics, comorbidities, and hospitalization outcomes between groups. We analyzed in-hospital mortality using Weibull regression and identified independent predictors via multivariable Cox proportional hazards models.

**Results:**

Of 112,348 bariatric surgery hospitalizations, 1,204 (1.1%) were in HIV-positive patients. HIV-positive patients were younger (51.0 vs. 56.1 years; *p* < 0.001), predominantly male (72.0% vs. 40.3%; *p* < 0.001), and had longer median lengths of stay (14.2 vs. 10.7 days; *p* < 0.001). Bariatric surgery rates among HIV-positive patients remained stable from 2017 to 2021 (*p* = 0.4). Compared with HIV-negative patients, those with HIV had higher prevalences of liver disease (11.7% vs. 1.7%; *p* < 0.001), renal disease (12.7% vs. 9.2%; *p* < 0.001), and a history of cancer (26.3% vs. 15.7%; *p* < 0.001). In unadjusted Weibull regression, HIV-positive status was associated with better in-hospital survival (*p* = 0.001), but after adjustment, HIV was not an independent predictor of mortality (HR 0.97; *p* = 0.843). Independent predictors of mortality across the entire cohort included advancing age (HR 1.04 per year; *p* < 0.001), sepsis (HR 3.36; *p* < 0.001), and liver disease (HR 1.70; *p* < 0.001).

**Conclusion:**

Bariatric surgery is safe for HIV-positive patients. Despite stable utilization, disparities persist. Further research is needed to improve access and assess long-term outcomes.

## Introduction

Bariatric surgery has been demonstrated to be a viable and safe option for HIV-positive patients [1]. Between 2004 and 2014, an estimated 4.4% of HIV-positive individuals with Class 3 obesity underwent bariatric surgery, highlighting its growing acceptance as a treatment option in this population [1, 2]. However, lingering hesitation persists, often driven by concerns about potential comorbidities and the influence of HIV on surgical outcomes.

One key source of uncertainty lies in the limited data on long-term outcomes and safety in this group. While some studies have shown that the procedure is both effective and safe, with sustained virologic suppression and significant weight loss, these studies often involve small sample sizes, outdated populations from over a decade ago, and relatively short follow-up periods [2–4].

This hesitation may be partly attributed to concerns about the pharmacokinetics of antiretroviral therapy (ART) following bariatric surgery. Procedures like sleeve gastrectomy and Roux-en-Y gastric bypass can alter the gastrointestinal tract, impacting drug absorption and potentially leading to subtherapeutic ART levels, which increases the risk of virologic failure and drug resistance [5]. This can be linked to increase in in-hospital comorbidities post-procedure and mortality in these patients.

The objective of this study is to evaluate the rates of in-patient comorbidities and in-hospital mortality among HIV-positive and HIV-negative patients undergoing bariatric surgery over the most updated national period with available data, providing a needed clarity on the safety and outcomes of this procedure in the HIV-positive patients.

## Material and Methods

### Data Source and Study Population

**Fig. 1:**
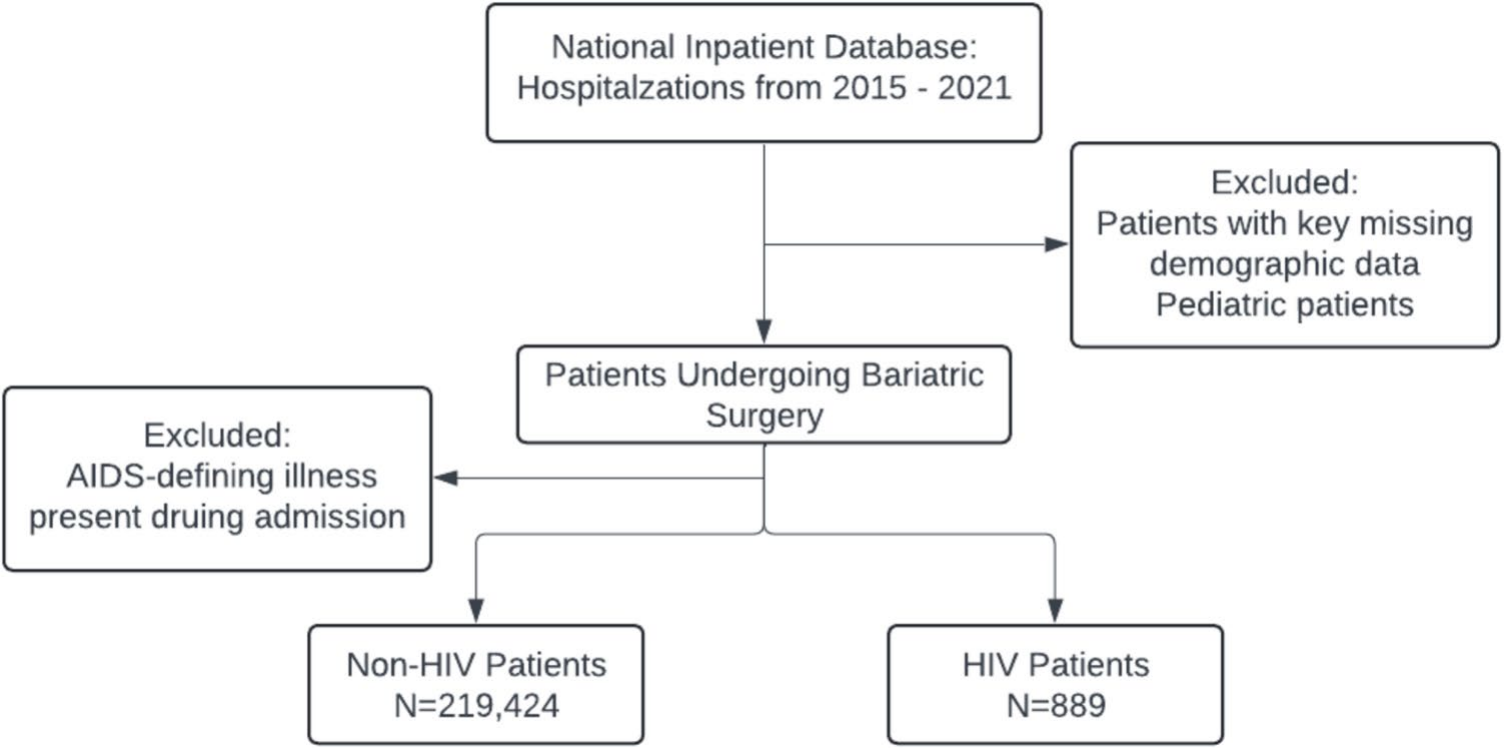
Flow diagram of study population selection. Abbreviations: HIV: Human Immunodeficiency Virus; AIDS: Acquired Immunodeficiency Syndrome

This study utilized data from the Nationwide Inpatient Sample (NIS), a comprehensive database of hospitalized patients in the United States. The analysis included records from 2015 to 2021 (Fig. 1). The NIS is a component of the Healthcare Cost and Utilization Project, managed by the Agency for Healthcare Research and Quality [6]. The unweighted dataset includes over 7 million hospitalizations annually, while the weighted dataset represents more than 35 million hospital stays per year. The database provides extensive information, including patient demographics (e.g., age, sex, race, median household income by ZIP code), hospital characteristics (e.g., urban or rural location, teaching or non-teaching status), expected payment sources, total hospitalization charges, length of stay, and diagnostic and procedural codes based on the International Classification of Diseases, 10th Revision, Clinical Modification (ICD-10-CM).

### Inclusion and Exclusion Criteria

Patients were included if they had ICD-10-CM codes indicating a primary or secondary diagnosis of HIV (Figure 1). Bariatric surgery was identified using ICD-10-PCS codes for procedures such as Roux-en-Y gastric bypass, gastric banding, and sleeve gastrectomy.

Exclusion criteria included hospitalizations with incomplete demographic or clinical data, such as missing age, sex, or mortality outcomes (Figure 1). Additionally, patients transferred to another acute-care facility were excluded to avoid duplication of cases. Patients <18 years old were also excluded. This ensured a well-defined cohort for assessing outcomes associated with bariatric surgery in patients with HIV.

### Covariates

Demographic and clinical characteristics were compared between HIV-positive and HIV-negative patients who underwent bariatric surgery. Covariates included age, gender, race/ethnicity, admission year, and median household income. Clinical factors analyzed included comorbidities such as diabetes, hypertension, liver and renal diseases, and HIV-related comorbidities. Hospitalization metrics, including length of stay and in-hospital mortality, were also assessed to evaluate differences in outcomes between the two groups.

### Statistical Analysis

Categorical variables were presented as counts and proportions, and differences tested using Pearson’s Chi-square test. Continuous variables were presented as mean (standard deviation), and differences between groups were tested using the t-test. The dataset was configured for survival analysis, where the time variable was the length of stay. Mortality during hospitalization was defined as the failure event. Patients discharged alive were treated as censored observations. A Weibull parametric survival regression model was applied to estimate the relationship between HIV status and in-hospital mortality. The Weibull distribution was chosen for its ability to model time-to-event data flexibly. After fitting the model, survival probabilities were estimated using a predict command. These predictions provided the probability of survival over the length of hospitalization for individual undergoing bariatric surgery based on their HIV status.

To identify significant predictors of survival, we conducted forward stepwise multivariate Cox regression analyses, which were adjusted for important demographic variables, comorbidities, and AIDS-related comorbidities. We included variables that were statistically significant at the bivariate level (partial regression (0.1) and partial elimination (0.05)) or were known to be clinically relevant. The results are presented as hazard ratios (HRs) with 95% confidence intervals (CIs), and statistical significance was defined as α = 0.05. All statistical analyses were conducted using Stata version 18.0 (StataCorp LP, College Station, TX, USA). According to the data user agreement, any individual table cell counts of 10 or fewer cannot be presented to preserve patient confidentiality. In such instances, data were suppressed and labeled as IS, information suppressed.

## Results

### General Characteristics

Among patients undergoing bariatric surgery, HIV-positive were generally younger, with a mean age of 51.0 years compared to 56.1 years in the non-HIV group (*p* < 0.001). A higher proportion of HIV-positive patients were male (72.0% vs. 40.3%, *p* < 0.001). The LOS was notably longer for HIV-positive patients, averaging 14.2 days compared to 10.7 days in the non-HIV group (*p* < 0.001), though the admission to surgery time was similar between groups (*p* = 0.05). Racial distribution differed significantly, with a higher proportion of Black patients and fewer White patients in the HIV-positive group (*p* < 0.001) (Table 1).

**Table 1:** Demographic and clinic patient characteristics

Variables	HIV Negative	HIV Positve	*P*-value
Age, mean ± SD (range in years)	56.12 ± 17.40 (0–90)	51.01 ± 11.66 (22–86)	<0.001
Female, n (%)	129,305 (59.74)	249 (28.04)	<0.001
Male, n (%)	90,037 (40.26)	639 (71.96)	<0.001
Time to admission to bariatric surgery, mean ± SD (range in days)	5.99 ± 9.49 (1–342)	6.98 ± 10.47 (1–134)	0.05
LOS, mean ± SD (range in days)	10.69 ± 15.73 (0–364)	14.19 ± 18.81 (0–171)	<0.001
Race, n (%)			<0.001
White	151,967 (71.61)	344 (39.63)	
Black	26,614 (12.54)	363 (41.82)	
Hispanic	22,074 (10.40)	120 (13.82)	
Asian	4,425 (2.09)	7 (0.81)	
Native American	1,165 (0.55)	4 (0.46)	
Other	5,979 (2.82)	30 (3.46)	
Median Household Income National, n (%)			<0.001
$1–24,999	59,206 (27.39)	337 (40.70)	
$25,000–34,999	58,240 (26.95)	205 (24.76)	
$35,000–44,999	54,940 (25.42)	180 (21.74)	
$45,000 or more	43,742 (20.24)	106 (12.80)	
Comorbidities, n (%)			
AMI	9,030 (4.12)	33 (3.71)	0.55
CHF	18,453 (8.41)	62 (6.97)	0.12
PVD	18,544 (8.45)	78 (8.77)	0.73
CEVD	4,509 (2.05)	22 (2.47)	0.38
COPD	21,273 (9.69)	92 (10.35)	0.51
Other Pulmonary Disease	26,301 (11.99)	132 (14.85)	0.01
Rheumatoid Disease	7,878 (3.59)	18 (2.02)	0.01
Dementia	5,124 (2.34)	7 (0.79)	0.002
HP/PAPL	4,838 (2.20)	23 (2.59)	0.44
Primary HTN	82,461 (37.58)	244 (27.45)	<0.001
Secondary HTN	76 (0.03)	0 (0.00)	0.579
DM2	42,897 (19.55)	129 (14.51)	<0.001
DM2 + Comorbidities	21,617 (9.85)	70 (7.87)	0.05
Liver Disease (All etiologies)	3,726 (1.70)	104 (11.70)	<0.001
RD	20,152 (9.18)	113 (12.71)	<0.001
PUD	7,955 (3.63)	23 (2.59)	0.10
Cancer	34,349 (15.65)	234 (26.32)	<0.001
Metastatic Cancer	25,399 (11.58)	96 (10.80)	0.47
Sepsis	36,549 (16.66)	176 (19.80)	0.012
AIDS	23 (0.01)	493 (55.46)	<0.001
Immunocompromised related comorbidities, n (%)			
Neutropenia	537 (0.24)	4 (0.45)	0.217
Cryptococcus	9 (0.00)	3 (0.34)	<0.001
Candidiasis	5,937 (2.71)	61 (6.86)	<0.001
Gonococcal Infection	2 (0.00)	0 (0.00)	0.928
Syphilis	14 (0.01)	2 (0.22)	<0.001
Histoplasmosis	24 (0.01)	4 (0.45)	<0.001
Pneumocystis	9 (0.00)	8 (0.90)	<0.001

*LOS* Length of Stay, *AMI* Acute Myocardial Infarction, *CHF* Congestive Heart Failure, *PVD* Peripheral Vascular Disease, *CEVD* Cerebrovascular Disease, *COPD* Chronic Obstructive Pulmonary Disease, *HP/PAPL* Hemiparesia/Paraplegia, *HTN* Hypertension, *DM* Diabetes Mellitus, *RD* Renal Disease, *PUD* Peptic Ulcer Disease

HIV-positive patients also exhibited significant differences in socioeconomic status and clinical comorbidities compared to non-HIV patients. A higher proportion of HIV-positive individuals fell into the lowest income bracket ($1–24,999; 40.7% vs. 27.4%, *p* < 0.001), with fewer in the highest income bracket ($45,000 or more; 12.8% vs. 20.2%, *p* < 0.001) (Table 1).

Regarding comorbidities, HIV-positive patients had higher rates of liver disease (11.7% vs. 1.7%, *p* < 0.001), renal disease (12.7% vs. 9.2%, *p* < 0.001), and cancer (26.3% vs. 15.7%, *p* < 0.001). Additionally, immunosuppression-related comorbidities were more frequent among HIV-positive patients, including cryptococcosis, candidiasis, syphilis, histoplasmosis, and pneumocystis (all *p* < 0.001). While some comorbidities, such as sepsis (19.8% vs. 16.7%, *p* = 0.012), were more common in the HIV-positive group, others, like dementia and primary hypertension, were less prevalent (Table 1).

### Bariatric Surgery Temporal Trends

**Fig. 2:**
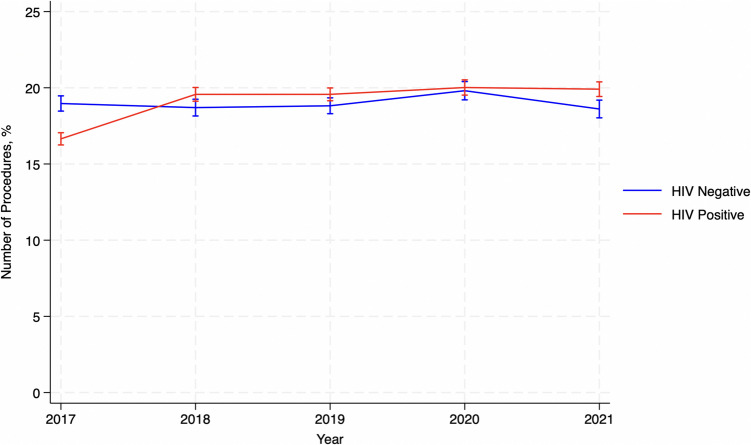
Distribution of bariatric surgery procedures in the last 5 years studied

The yearly trends of the bariatric surgery population included in this analysis reveal a relatively stable distribution of cases from 2017 to 2021. Among the patients included in this study, in HIV-negative patients, the proportion of admissions ranged from 18.6% in 2021 to 19.8% in 2020. Similarly, HIV-positive patients showed consistent proportions, ranging from 16.6% in 2017 to 20.0% in 2020. These trends suggest a steady rate of bariatric surgery utilization over recent years, with no significant variation in the distribution between HIV-positive and HIV-negative groups (*p* = 0.4) (Fig. 2).

**Table:** 

Variables	HIV Negative	HIV Positive	*P*-value
Admission Year, n (%)			0.4
2017	41,623 (18.97)	148 (16.65)	
2018	41,025 (18.70)	174 (19.57)	
2019	41,304 (18.82)	174 (19.57)	
2020	43,478 (19.81)	178 (20.02)	
2021	40,830 (18.61)	177 (19.91)	

## Predicted In-Hospital Mortality

**Fig. 3:**
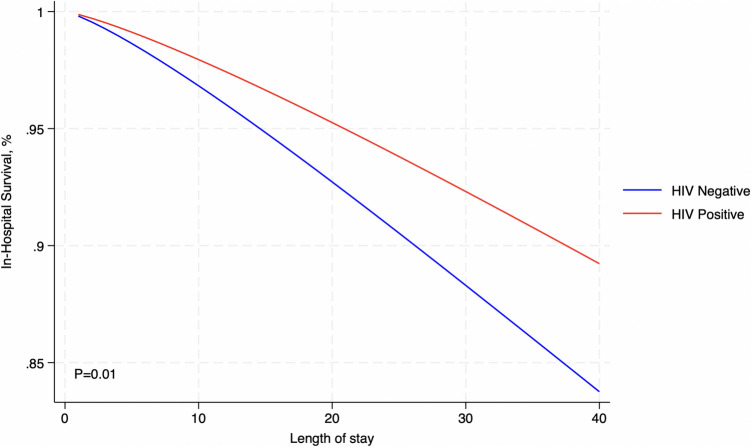
Predicted in-hospital mortality between the groups

In-hospital mortality rates showed a lower survival function among HIV-positive patients compared to HIV-negative patients as the LOS increased (P<0.01) (Figure 3). At 10 days, the survival function was slightly lower for HIV-positive patients (0.97 vs. 0.98). This disparity became more pronounced with longer stays, with survival functions of 0.93 vs. 0.95 at 20 days, 0.88 vs. 0.92 at 30 days, and 0.84 vs. 0.89 at 40 days (Figure 3).

**Table:** 

Length of Hospital Stay	HIV Negative				HIV Positive			
	Number at risk	Events	Survival Function	95% CI	Number at risk	Events	Survival Function	95% CI
10	78,806	3,906	0.97	0.96 - 0.98	387	10	0.98	0.98 - 0.98
20	30,136	2,667	0.93	0.92 - 0.94	182	12	0.95	0.95 - 0.95
30	13,706	1,206	0.88	0.88 - 0.88	101	3	0.92	0.92 - 0.92
40	7,306	527	0.84	0.84 - 0.84	61	5	0.89	0.89 - 0.89

### Predictors of In-Hospital Mortality

An univariate and multivariate Cox regression analysis, adjusting for confounders to better elucidate the impact of clinical and demographic factors, was conducted. Variables were included in the multivariate model based on their significance in the univariate analysis (*p* < 0.05), to avoid overfitting (Table 2).

**Table 2:** Predictors of in-hospital mortality in HIV-patients undergoing bariatric surgery

Variables	Univariate			Multivariate		
	HR	95% CI	P-value	HR	95% CI	*P*-value
HIV	0.65	0.46 - 0.92	0.01	0.97	0.68 - 1.37	0.843
Year	0.99	0.97 - 1.00	0.06	0.99	0.98 - 1.00	0.196
Age	1.04	1.03 - 1.04	<0.001	1.04	1.03 - 1.04	<0.001
Female sex	1.06	1.02 - 1.11	<0.001	1.03	0.98 - 1.07	0.248
Race						
White	-	-	-	-	-	-
Black	0.71	0.67 - 0.76	<0.001	0.84	0.79 - 0.90	<0.001
Hispanic	0.69	0.64 - 0.75	<0.001	0.89	0.83 - 0.97	0.005
Asian	0.73	0.63 - 0.84	<0.001	0.73	0.63 - 0.85	<0.001
Native American	0.96	0.74 - 1.24	0.745	-	-	-
Other	0.74	0.64 - 0.84	<0.001	0.89	0.78 - 1.02	0.09
Comorbidities						
HTN	0.54	0.51 - 0.58	<0.001	0.71	0.67 - 0.76	<0.001
DM2	0.78	0.73 - 0.83	<0.001	0.87	0.81 - 0.93	<0.001
AMI	2.2	2.07 - 2.35	<0.001	1.35	1.27 - 1.44	<0.001
CHF	2.3	2.20 - 2.41	<0.001	1.15	1.09 - 1.21	<0.001
PVD	2.77	2.65 - 2.89	<0.001	1.79	1.71 - 1.88	<0.001
Stroke	1.74	1.61 - 1.88	<0.001	1.24	1.14 - 1.34	<0.001
COPD	1.92	1.83 - 2.02	<0.001	1.23	1.17 - 1.29	<0.001
Other Pulmonary Disease	1.36	1.29 - 1.43	<0.001	1.05	0.99 - 1.11	0.077
Rheumatoid Disease	1.07	0.97 - 1.17	0.19	-	-	-
Dementia	1.95	1.79 - 2.12	<0.001	1.08	0.99 - 1.18	0.083
HP/PAPL	0.84	0.80 - 0.89	<0.001	0.90	0.85 - 0.96	0.001
Liver Disease	1.68	1.52 - 1.87	<0.001	1.70	1.53 - 1.89	<0.001
RD	1.39	1.36 - 1.43	<0.001	1.05	1.02 - 1.07	0.001
PUD	1.3	1.20 - 1.40	<0.001	1.14	1.06 - 1.24	0.001
Cancer	0.98	0.95 - 1.01	0.168	-	-	-
Metastatic Cancer	1.02	1.01 - 1.03	<0.001	1.06	1.05 - 1.07	<0.001
Sepsis	4.54	4.34 - 4.76	<0.001	3.36	3.20 - 3.53	<0.001
AIDS	0.95	0.89 - 1.01	0.073	-	-	-
AIDS-related Comorbidities						
Neutropenia	1.23	0.94 - 1.61	0.12	-	-	-
Cryptococcus	1.75	0.66 - 4.66	0.26	-	-	-
Candidiasis	0.76	0.70 - 0.83	<0.001	0.67	0.62 - 0.74	<0.001
Pneumocystis	0.32	0.04 - 2.25	0.25	-	-	-

*LOS* Length of Stay, *HTN* Hypertension, *DM* Diabetes Mellitus, *AMI* Acute Myocardial Infarction, *CHF* Congestive Heart Failure, *PVD* Peripheral Vascular Disease, *COPD* Chronic Obstructive Pulmonary Disease, *HP/PAPL* Hemiparesia/Paraplegia, *RD* Renal Disease, *PUD* Peptic Ulcer Disease

HIV status, which was significant in the univariate analysis (HR: 0.65, 95% CI: 0.46–0.92, *p* = 0.01), was not significant in the multivariate model (HR: 0.97, 95% CI: 0.68–1.37, *p* = 0.843), after adjusting for demographic and important clinical variables (Table 2).

Other notable findings included the impact of race, with Black patients showing lower adjusted mortality risk (HR: 0.84, 95% CI: 0.79–0.90, *p* < 0.001) compared to White patients. Similarly, Hispanic patients had a reduced mortality risk (HR: 0.89, 95% CI: 0.83–0.97, *p* = 0.005), while Asian patients maintained a significant protective effect (HR: 0.73, 95% CI: 0.63–0.85, *p* < 0.001) (Table 2).

Significant clinical predictors in the multivariate model included age (HR: 1.04 per year, 95% CI: 1.03–1.04, *p* < 0.001) and key comorbidities such as sepsis (HR: 3.36, 95% CI: 3.20–3.53, *p* < 0.001), Peripheral Venous Disease (HR: 1.79, 95% CI: 1.71–1.88, *p* < 0.001), Acute Myocardial Infarction (HR: 1.35, 95% CI: 1.27–1.44, *p* < 0.001), and Liver Disease (HR: 1.70, 95% CI: 1.53–1.89, *p* < 0.001) (Table 2).

## Discussion

This study provides current, most updated analyses of in-hospital outcomes for HIV-positive patients undergoing bariatric surgery in the United States, utilizing data from the Nationwide Inpatient Sample between 2015 and 2021. Our findings confirm that bariatric surgery remains a viable option for HIV-positive patients, with no significant impact of HIV on in-hospital mortality when adjusting for comorbidities and demographic factors. This aligns with previous findings from Sharma et al. (2018), which reported no increased mortality in HIV-positive patients undergoing bariatric surgery between 2004 and 2014​ [2].

Our study highlights that while HIV status is not an independent predictor of in-hospital mortality, comorbidities such as sepsis, liver disease, and cardiovascular comorbidities play a significant role. These findings are consistent with Hui et al. (2021), which identified sepsis as a major contributor to mortality following bariatric surgery, emphasizing the importance of proactive perioperative management in high-risk populations [7, 8]. In contrast to earlier work that did not include predictors like liver disease or PVD in multivariate models, our analysis provides a nuanced understanding of factors driving mortality.

Our findings highlight the pathophysiologic drivers of HIV in patients undergoing bariatric surgery. Despite their younger age in HIV, this population had a disproportionately higher burden of liver disease, renal disease, and sepsis [9]. Prior literature has described ART-associated hepatotoxicity and increased rates of nonalcoholic fatty liver disease and viral hepatitis co-infection in HIV-positive individuals, all of which may contribute to multiorgan dysfunction [10]. Similarly, persistent immune dysregulation, even in the setting of controlled viral load, may increase vulnerability to sepsis [10]. Cardiovascular risks may also be elevated due to chronic inflammation and ART-related metabolic effects [9, 10]. These mechanisms likely contribute to the elevated comorbidity profile observed in our cohort and underscore the importance of tailoring perioperative risk assessment in them.

Racial and socioeconomic disparities in bariatric surgery outcomes were evident in our findings. Black and Hispanic patients had lower adjusted mortality risks compared to Whites, aligning with Chen et al. (2023), which highlighted racial differences in postoperative outcomes [10]. These results contrast with earlier studies, such as Nguyen et al. and Patel et al., which reported higher in-hospital mortality among non-Hispanic Black patients, particularly in low-volume hospitals, likely due to disparities in healthcare delivery [11]. Robitson et al. also noted that Black patients often present with higher BMI and more comorbidities, complicating recovery [12, 13]. One possible explanation for our differing findings is that our cohort, drawn from high-volume academic centers, may reflect improved access to care, enhanced multidisciplinary support, and standardized HIV management protocols, which may mitigate disparities seen in prior studies.

A unique aspect of our study is the analysis of HIV-specific comorbidities, such as candidiasis and cryptococcosis, which were more prevalent in HIV-positive patients but did not independently predict mortality. This supports findings from Kaip et al. (2022), which demonstrated that ART efficacy is generally maintained post-surgery, with most patients achieving sustained virologic suppression​ [14]. However, individual variability in ART pharmacokinetics, as highlighted by MacBrayne et al. (2014), remains a concern, particularly for procedures like Roux-en-Y gastric bypass that alter gastrointestinal absorption​ [5, 14].

### Limitations

This study has several limitations inherent to the use of the NIS database. First, the NIS is an administrative database and relies on ICD-10 coding, which may be subject to misclassification or coding errors. Second, it captures only inpatient encounters and lacks outpatient follow-up, thereby limiting the ability to assess long-term outcomes, readmissions, and post-discharge complications. Related to this issue, most of the patients included are treated at large referral centers, which often manage individuals with higher complexity, potentially overrepresenting certain findings such as comorbidity burden or prolonged hospital stays.

Third, granular clinical details, such as CD4 counts, viral load, ART regimen type, or nutritional status, are not available, which prevents a more comprehensive analysis of how HIV severity and treatment adherence impact outcomes. The NIS also lacks procedure-specific details such as the type of bariatric surgery performed, which limits stratified comparisons between sleeve gastrectomy and Roux-en-Y gastric bypass, each of which may have different risk profiles, especially in patients with altered ART pharmacokinetics. Additionally, there is no ability to determine whether comorbidities developed postoperatively or were preexisting, limiting our capacity to assess postoperative complications or differentiate between chronic and acute disease processes. Finally, socioeconomic and hospital-level variables such as access to multidisciplinary care or regional variations in HIV management protocols are not fully accounted for in the database.

## Conclusion

This study demonstrates that bariatric surgery is safe in HIV patients in the perioperative period and identifies key factors influencing in-hospital mortality. We found that sepsis, liver disease, and cardiovascular comorbidities significantly increase mortality risk, underscoring the importance of vigilant perioperative management. Racial and socioeconomic disparities persist, with Black and Hispanic individuals being more prevalent in the HIV group, exhibiting lower adjusted mortality risks but facing greater overall socioeconomic disadvantages. Notably, HIV-specific comorbidities did not independently predict mortality, highlighting the protective role of optimized ART management. Although procedural rates remained stable in both groups, it remains unclear whether utilization should be increasing given advances in surgical safety and the burden of obesity in HIV. Further research is essential to address disparities, improve long-term outcomes, and expand access of obesity treatments such as bariatric surgery for people living with HIV.

### Future Directions

Future research should focus on the long-term outcomes of bariatric surgery in HIV-positive patients. These include sustained weight loss, long-term survival, and improvement in obesity-related comorbidities such as diabetes and cardiovascular disease. Additionally, studies should evaluate ART pharmacokinetics over time, changes in immune function, and the impact of bariatric surgery on virologic suppression. Prospective cohort studies with longer follow-up and greater clinical granularity are essential to better understand how bariatric surgery affects quality of life, medication adherence, and overall HIV management in the years following surgery.

## Data Availability

No datasets were generated or analysed during the current study.
